# Searching for the origins of musicality across species

**DOI:** 10.1098/rstb.2014.0094

**Published:** 2015-03-19

**Authors:** Marisa Hoeschele, Hugo Merchant, Yukiko Kikuchi, Yuko Hattori, Carel ten Cate

**Affiliations:** 1 Department of Cognitive Biology, Vienna, Austria; 2 Instituto de Neurobiologia, UNAM, Campus Juriquilla, Santiago de Querétaro, Mexico; 3 Institute of Neuroscience, Newcastle University Medical School, Newcastle upon Tyne, UK; 4 Primate Research Institute, Kyoto University, Kyoto, Japan; 5 Institute of Biology, Leiden University, Leiden, The Netherlands; 6 Leiden Institute for Brain and Cognition, Leiden University, Leiden, The Netherlands

**Keywords:** musicality, music perception, evolution of music, animal models, comparative studies

## Abstract

In the introduction to this theme issue, Honing *et al.* suggest that the origins of musicality—the capacity that makes it possible for us to perceive, appreciate and produce music—can be pursued productively by searching for components of musicality in other species. Recent studies have highlighted that the behavioural relevance of stimuli to animals and the relation of experimental procedures to their natural behaviour can have a large impact on the type of results that can be obtained for a given species. Through reviewing laboratory findings on animal auditory perception and behaviour, as well as relevant findings on natural behaviour, we provide evidence that both traditional laboratory studies and studies relating to natural behaviour are needed to answer the problem of musicality. Traditional laboratory studies use synthetic stimuli that provide more control than more naturalistic studies, and are in many ways suitable to test the perceptual abilities of animals. However, naturalistic studies are essential to inform us as to what might constitute relevant stimuli and parameters to test with laboratory studies, or why we may or may not expect certain stimulus manipulations to be relevant. These two approaches are both vital in the comparative study of musicality.

## 1. Introduction

Honing *et al.* [1] suggest that the origins of musicality—the capacity that makes it possible for us to perceive, appreciate and produce music—can be pursued productively by searching for components of musicality in other species. Perhaps the most obvious starting point in this endeavour is the examination of animal responses to music. In 1984, Porter & Neuringer [2] were the first to conduct an experiment from this perspective by training pigeons (*Columba livia*) to discriminate the music of different composers. The authors used an operant paradigm, where pigeons received a food reward after pecking one of two discs during presentation of excerpts from several Bach pieces for organ and Stravinsky's *Rite of spring*. Pigeons were trained to respond to the left disc during Bach, and a right disc during Stravinsky excerpts. With time, the pigeons learned this discrimination. Once the pigeons were making few errors, they were presented with novel excerpts from the same composers, and similar excerpts from other composers. The pigeons generalized to all of these novel stimuli through their responses to the two choice discs in a way that mirrored that of human participants.

A more recent study was performed using a similar operant paradigm with carp (*Cyprinus carpio*) using blues and classical stimuli and found comparable results [3]; after initial training with a small set of blues and classical music stimuli, carp were able to correctly classify stimuli from these genres that they had never heard before. How can we interpret the fact that distantly related animal species have human-like boundaries for the categorization of such complex auditory stimuli? Moreover, what can we learn from such studies?

## 2. Key problems in studying biomusicology

The genre classification performance of pigeons and carp has an analogue in studies on visual categorization. In their seminal study, Hernnstein & Loveland [4] successfully trained pigeons to discriminate photos that contained humans from photos that did not, with all photos exhibiting considerable variability. Subsequent debate has centred on whether the pigeons were detecting humans or simply using local features (e.g. large flesh-coloured area) to solve the task [5]. Similarly, pigeons and carp in the music categorization tasks may use specific local features (e.g. presence of absence of a specific frequency) rather than use global or abstract features to solve the task. One way to determine what features are controlling the behaviour in each species would be to present altered stimuli that are missing some of the features from the rich training stimuli or present some features of the rich stimuli in isolation.

A second, and equally important issue, is motivational. Aside from the music categorization abilities demonstrated in pigeons and carp, do they have any music preferences? We know that chimpanzees prefer at least some types of music to silence, as they spent more time close to a speaker when music was being played than when it was not [6]. But another primate species not as closely related to humans, the cotton-top tamarins (*Saguinus oedipus*), showed the opposite result [7]. Several other studies have looked at animal musical preferences using similar place preference paradigms. Chiandetti & Vallortigara [8] put newborn chicks (*Gallus gallus*) in an environment with consonant music playing on one side, and dissonant music on the other. They found that the chicks spent more time on the side with consonant music. When McDermott & Hauser [9] presented cotton-top tamarins with consonant stimuli in one arm of a Y-maze and dissonant stimuli in the other arm, the animals showed no preference. Western adults confronted with similar contingencies spent more time listening to consonant than to dissonant sounds. Although such a preference paradigm suggests that a feature such as consonance and dissonance is relevant to a given species, it tells us little about the mechanisms underlying any preferences. The human preference for consonance over dissonance is at least partially based on the physical properties of sound and is evident across many cultures [10]. It is equally important to ascertain the factors contributing to music-related preferences in non-human species.

Another problem is the selection of appropriate species for study. Taking a traditional laboratory approach, it is possible to take virtually any species with sound-sensing capacity and rudimentary learning capacities and measure its physiology and train it to discriminate different sounds. But which species are relevant for biomusicology? One approach is to study species based on shared ancestry. Species that are closely related to humans are likely to share some of our abilities, and are therefore good models for experiments that would be difficult to conduct in humans. For traits that are not shared among closely related species, it is easier to pinpoint the differences in underlying mechanisms. Species that are more distantly related sometimes share traits without sharing a common ancestor with those traits. By examining the evolutionary convergence between these non-related species, we can identify biological constraints or mechanisms required for that trait and the selection pressures giving rise to it. For example, some of the traits that are considered highly relevant for biomusicology to date are vocal learning and entrainment. Vocal learning involves the ability to produce vocalizations based on auditory input, and entrainment involves the ability to synchronize movements with an external stimulus (usually sound). Both traits are uncommon in non-human species but shared across some unrelated species, so their study could provide clues to their nature and possible interaction [11,12]. Thus, both approaches, examining closely related and distantly related species, can be quite useful for probing the biological basis of musicality.

Thus, we need to consider the perceptual abilities of animals, their natural preferences, as well their similarity to humans in terms of phylogeny or shared traits. This task necessitates the combined fruits of traditional laboratory studies with artificial stimuli and more naturalistic studies.

## 3. Traditional laboratory studies

There is much debate about the relative utility of naturalistic and artificial laboratory studies. Proponents of naturalistic studies argue that training animals to perform ‘unnatural’ behaviours, or using stimuli that differ markedly from those in their natural environment, does not constitute an appropriate comparison for human behaviours that emerge without training. They also point out that artificial stimuli can result in underestimation of animal abilities. Proponents of laboratory research note that experimental control and systematic comparisons across species may reveal underlying abilities and potential that are not apparent in natural behaviour. As a result, laboratory studies can shed light on the presence of cognitive abilities that support such behaviour as well as their biological basis. Both sides have valid insights about the limitations of the other approach, but they fail to appreciate its strengths and the utility of combined approaches. For example, much has been gained from studying behavioural and neural processes in various species in response to artificial auditory patterns presented in laboratory settings.^1^

### (a) Rhythm

The processing of auditory rhythms—both the underlying pulse or beat and the organization of the beats into repetitive groups—relies on basic features of the auditory system. The use of operant conditioning procedures has revealed greater temporal sensitivity in birds than in humans [13] when evaluating perceptual differences among brief stimuli. In another study [14], pigeons successfully learned to differentiate two metrical patterns (8/4 versus 3/4) and to transfer the discrimination to different tempos, but their learning did not generalize to metrical patterns in a different timbre. In addition, they had difficulty differentiating rhythmic sequences from random sequences. European starlings, however, learned to differentiate rhythmic from non-rhythmic sequences and showed a broader range of generalization [15].

The use of neurophysiological (e.g. functional magnetic resonance imaging, fMRI) measures has revealed that the neural substrates of sequencing and timing behaviours overlap with those related to human music perception and performance (see [16] for a review) and that the motor corticobasal ganglia–thalamocortical circuit (mCBGT) plays an important role [17–19]. Not only are trained rhesus macaques (*Macaca mulatta*) capable of interval timing and motor sequencing tasks performed by humans [20–24], but they also show similar neural activation in mCBGT in both sequential [25,26], and single interval timing tasks [27–29]. These results suggest that the role of mCBGT in auditory rhythm processing is shared across these two species of primates. Uncovering findings such as these was only possible with the use of artificial laboratory probing of the limits of the perceptual systems of these two species.

### (b) Timbre

Timbre perception has not received much attention in the animal literature, but laboratory studies have begun to provide some insights. As with temporal intervals, avian species seem to detect more fine-grained timbral differences than humans do [30–32]. Timbre is considered a surface [33] feature, because humans recognize the same musical patterns, regardless of the timbre of presentation (e.g. vocal, piano, flute). This ability to generalize across timbres is reportedly present from the newborn period [34]. In one study, humans' responses to chords readily generalized across timbres, but that was not the case with black-capped chickadees (*Poecile atricapillus*) [35]. Zebra finches exhibit generalization across timbres [36,37]. When trained to discriminate between two words produced by male (or female) speakers, they showed generalization across speaker gender (i.e. fundamental frequency and spectral differences). Clearly, more research is needed to clarify the nature and extent of timbre generalization across species.

### (c) Pitch

The pitch of a sound is typically based on the fundamental frequency of that sound [38], although human listeners can perceive the pitch of a sound in which the fundamental frequency is missing [39]. The ability to perceive the so-called missing fundamental is present in infants as young as three months of age [40] and is also demonstrable in cats (*Felis catus* [41]), rhesus monkeys [42] and starlings [43]. The assumption is that this ability is shared across species, but its generality has not been established empirically.

Listeners sometimes evaluate fundamental frequency or pitch in an absolute manner, as when musicians with absolute pitch correctly name the pitch class of musical notes (e.g. 440 Hz as A) [44] or non-musicians distinguish the original pitch level of highly familiar recorded music from versions that have been shifted by one or two semitones [45]. In general, birds are superior to mammals at detecting absolute pitch ([46–48], but see [49]). In most cases, however, human listeners focus on relations among pitches rather than absolute pitch levels while listening to music. In musical contexts, moreover, human listeners exhibit octave generalization, perceiving the similarity of notes that are one or more octaves apart [50,51].

The evidence for octave generalization in non-human species is both limited and controversial. Blackwell & Schlosberg [52] claimed that rats (*Rattus norvegicus*) generalized from training stimuli in one octave to test stimuli in another octave. However, there are alternative explanations of the findings, because the stimuli may have contained harmonics that provided common cues across octaves [50]. Suggestive evidence for octave generalization comes from a bottlenose dolphin (*Tursiops truncatus*) that mimicked sounds outside of her vocal range by reproducing them an octave apart from the original [53]. Interestingly, rhesus monkeys trained to differentiate melodies in a same–different task responded to octave-transposed melodies as ‘same’ for Western tonal, but not atonal melodies [54].

To date, there is no evidence of octave generalization in avian species. Cynx [55] trained starlings to discriminate between two tones, and then tested whether they generalized this discrimination to the octave. They did not. The failure of human listeners to exhibit octave generalization on the same task [56] called the starling findings into question. In a similar operant training task, humans exhibited octave generalization [56], but an adaptation of the task for black-capped chickadees revealed no octave generalization [57]. The available evidence is consistent with the absence of octave generalization in birds, but more laboratory research is needed with a wider range of species before the question can be resolved definitively.

With regards to relative pitch, several studies have found that non-human animals could be trained to discriminate among chords (i.e. simultaneous combinations of tones): European starlings [58], Java sparrows (*Lonchura oryzivora*; [59]), Japanese monkeys (*Macaca fuscata*; [60]), pigeons [61] and black-capped chickadees [35,62]. All of these studies ensured the animals were not simply memorizing the absolute properties of the sounds by presenting novel stimuli with identical or similar relative pitch properties but different absolute pitches. All species were able to transfer what they learned to these novel stimuli.

Evidence of relative pitch processing with sounds presented sequentially rather than simultaneously is less promising. Starlings, brown-headed cowbirds (*Molothrus ater*), and northern mockingbirds (*Mimus polyglottos*) were trained to discriminate ascending from descending note patterns [63–67]. However, they failed to generalize these patterns to novel pitch levels when the altered patterns were outside the training range, although they could quickly learn to do so. In general, it appeared that the birds encoded both absolute and relative pitch information in discriminating the patterns but depended more on absolute information. Another set of studies trained zebra finches and black-capped chickadees to discriminate sets of pitches based on either their pitch ratios (i.e. relative pitch) or their absolute frequencies. Both species learned the discrimination more quickly when there was a simple relative pitch rule that they could use, although the discrimination was quite difficult for the birds in comparison with learning a simple absolute pitch rule. In short, these birds can engage in relative pitch processing although they rely primarily on the absolute pitch of sounds [68,69]. In one study, a bottlenose dolphin learned to respond to descending pitch contours, and after extensive training, generalized that response to descending pitch contours regardless of the component pitches [70].

In general, non-human species recognize the relative pitch patterns of single chords more readily than those of note sequences. Three factors may be implicated. First, the component frequencies of chords give rise to qualities such as sensory consonance and dissonance [71] that contribute to their distinctiveness. Second, chords, as single events, pose fewer memory demands than sequences of notes. Third, there are suggestions that harmonic (simultaneous) pitch ratios are processed at early stages of the auditory cortical pathway in rhesus macaques [72]. As a result, the pitch ratios of chords or simultaneously presented notes may be processed more automatically and therefore compared more readily than the pitch ratios of melodic sequences.

### (d) High-order acoustic patterns

Building on the foundations of the auditory system and interval timing is the perception of grouping. Gestalt psychologists noted long ago that a group of visual or auditory elements has qualities that are more than the sum of its parts. A repeated series of tones of equal frequency and amplitude, with one tone having longer duration than the others, is perceived as an iambic pattern in which the long sound marks the end of a sound unit [73]. A repeated series of tones in which one tone has higher frequency or amplitude than the others is perceived as a trochaic pattern in which the higher pitched or louder sound marks the beginning of the sound unit [73]. This type of patterning is common in music as well as speech and young infants seem to spontaneously recognize trochaic patterns [74]. Rats seem to group tones according to trochaic, but not iambic rules [75], which indicates that such grouping abilities are not exclusive to human listeners. Further research is needed to explore the nature of auditory grouping abilities across species.

The ability to perceive higher-order temporal patterns in a stream of sounds is relevant to speech as well as music perception. The perception of speech prosody, or the melody of speech, is relevant to music perception. In many languages, for example, statements end with a falling terminal pitch contour, and yes/no questions end with a rising terminal pitch contour [76]. Different languages also have different prosodic patterns [77]. Tamarins [78], rats [79,80] and Java sparrows [81] have shown the ability to discriminate between spoken sentences in different languages and to generalize this discrimination to novel sentences. Zebra finches use pitch, duration and amplitude to discriminate prosodic patterns, and they can generalize specific prosodic patterns of speech syllables to novel syllables [36]. Further exploration of non-human species' sensitivity to melodic aspects of speech may be a fruitful approach to the study of some aspects of musicality.

### (e) Criticisms of laboratory studies

When non-human animals are trained to discriminate auditory patterns, they typically take a lot longer to learn the task than their human counterparts. A critic may ask, for example, whether a bird trained for hundreds of trials to discriminate chords can really be compared with a human who discriminates the chords without training or with minimal training. That situation does not negate the value of comparisons of music perception in human and non-human listeners. Although human listeners may require little training for specific tasks, they have had years of exposure to music and have a wealth of implicit musical knowledge. Moreover, the ability of non-human listeners to perform certain tasks, even after extensive training, can provide insights into the mechanisms underlying that ability.

Consider the studies of interval timing in rhesus macaques and humans. As the interaction between the auditory stimulus and required motor output becomes more complex, monkeys' performance lags increasingly behind that of humans. In one study, monkeys and humans were required to tap on a push-button to produce six isochronous intervals in a sequence. An auditory stimulus was present to guide tapping during the first three taps but not the last three, which required internal timing based on the preceding auditory stimulus or taps [20,82]. Although monkeys produced rhythmic movements with appropriate tempo matching, their movements lagged by approximately 250 ms after each auditory stimulus, even after long periods of training (close to a year; [20]). In contrast, humans easily perform the same task, with no training, showing stimulus movement asynchronies approaching zero or with negative values [20,83]. Such differences in two closely related species make it possible to predict that the mCBGT may have subtle, but critical differences that evolved in order to process complex auditory information and use it in a predictive fashion to control the temporal and sequential organization of movement, as recently stated in the gradual audiomotor evolution hypothesis [84].

Even if humans and monkeys had comparable experience with the stimuli in such experimental tasks, which they do not, both have very different interpretations of the experimental context and the experimenter's intentions, even where efforts have been made to minimize differences in training requirements and outcomes [35,49,57,62].

### (f) Conclusions

Overall, the aforementioned evidence indicates the enormous potential of laboratory studies of some components of musicality with non-human species. Operant conditioning studies have the potential to reveal skills that are not part of an animal's natural repertoire. Animals' performance in these tasks is deeply rooted in the limitations and adaptive plasticity of their nervous system [85]. By using these animals as models, we can gain information about the neural activity (e.g. through electrophysiological recordings) as well as manipulations (e.g. pharmacology, electric-stimulation, optogenetics) that can alter brain mechanisms and corresponding behaviour, facilitating our understanding of the neural underpinnings of musicality in humans.

## 4. Importance of natural behaviour

Laboratory experiments with artificial stimuli have been helpful in revealing perceptual skills and perceptual–motor coordination in non-human species. It is possible, however, that their use may lead to underestimates of ability. One alternative or supplementary approach is to use biologically relevant stimuli in laboratory studies. Another is to study music-like features in the natural behaviour (e.g. vocalizations) of animals.

### (a) Incorporating naturalistic stimuli into experimental work

As noted, laboratory research with artificial stimuli revealed that birds focus on absolute aspects of pitch rather than relative pitch [63–69], but evidence from field studies suggests otherwise. For example, fieldwork with black-capped chickadees has shown that they produce a simple two-note tonal song that can be sung at different absolute pitches, but maintains its relative pitch ratio [86]. Moreover, this relative pitch ratio is produced more accurately by dominant males [87], and accurately produced song pitch ratios are preferred by females [88]. These findings prompted laboratory research on this issue with chickadees [89]. Chickadees were trained to discriminate pitch ratios presented at different absolute frequencies, and made use of this relevant song pitch ratio. An experimental group was trained to respond to the pitch ratio from chickadee song and not to respond to two non-chickadee-song pitch ratios. A control group was trained to respond to a non-chickadee-song pitch ratio and not respond to two different non-chickadee-song pitch ratios. The chickadees that were required to identify the pitch ratio of their song learned the task more quickly than the control group, suggesting that it was easier for the chickadees to learn to discriminate the natural song pitch ratio than other pitch ratios [89]. A related study showed that starlings that were trained to discriminate conspecific vocalizations were able to maintain that discrimination even when the songs were transposed (i.e. pitches changed, but pitch relations preserved) [90], raising the possibility that absolute pitch processing has priority over relative pitch processing only with stimuli lacking in ecological validity.

There are parallels in the realm of rhythm perception. Although pigeons have difficulty with rhythm perception tasks involving artificial stimuli [14], the natural coo vocalizations of pigeons and doves, neither of which are vocal learners, are rhythmic. The collared dove produces a coo that consists of five elements of different duration: three notes separated by two silences [91]. Playback experiments in the field show that replacing the second or third note of the coo by silence caused little change in the behavioural response to the coo. When the removed note was not replaced by silence, shortening the duration of the coo, or when the pauses before and after the second note were reversed, the response was significantly reduced. This suggested a sensitivity to the overall rhythmic structure of the coos [92]. Although rhythm perception in doves may be closely tied to properties of their natural coos, it is important to explore sensitivity to rhythms in patterns that share at least some properties with natural vocalizations. In another study, zebra finches were trained to discriminate conspecific songs and subsequently tested with novel versions that altered amplitude, fundamental frequency or duration [93]. Although performance decreased substantially with changes in amplitude or fundamental frequency, it was maintained with duration changes of over 25%, well beyond zebra finches' reported sensitivity to temporal changes [13]. The implication is that the rhythmic patterning is particularly important for pattern classification in zebra finches.

### (b) Music-like features in natural behaviour

**Figure 1.**
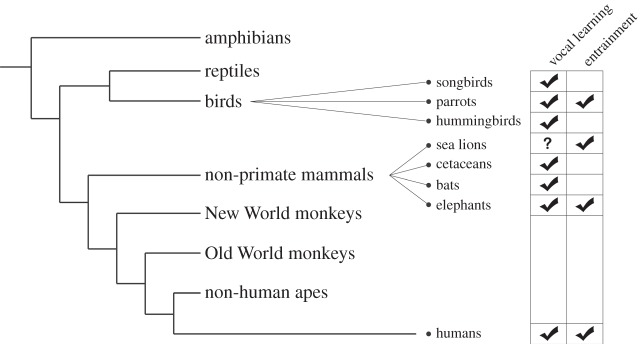
Species with vocal learning and entrainment abilities and their relationship in a phylogenetic tree.

The two best known features of musicality found in distantly related species are vocal learning and entrainment (see glossary, table 1). There are suggestions that the two abilities are related [94]. To date, the species that have been shown to exhibit both vocal learning and entrainment are distantly related to humans. Figure 1 shows the relatedness of various vertebrate species, indicating which have vocal learning and entrainment abilities.

**Table 1.** Glossary of relevant musical terms.

term	definition
beat	the underlying pulse, or unit of time, in music
entrainment	the ability to perceive a beat in music and align bodily movement with it
melody	a sequence of tones defined by its pitch patterning and rhythm
meter	the recurring pattern of stressed and unstressed beats in music
musicality	the capacity that underlies the human ability to perceive, appreciate, and produce music
pitch	a perceptual attribute related to the fundamental frequency that enables comparisons of sounds as higher or lower
prosody	rhythm, loudness, pitch, and tempo of speech
rhythm	a non-random repetitive temporal auditory pattern
timbre	the quality of musical sound that distinguishes different sound sources such as voices and specific musical instruments
vocal learning	long-term modification of vocal production by imitation

The greatest focus has been on vocal learning, with much greater concern for its relevance to language acquisition [95] than to musicality. However, vocal learning is also relevant to music production. For example, consider the extensive research of Nicolai [96,97] on vocal learning in the bullfinch (*Pyrrhula pyrrhula*), a songbird. Although bullfinches normally learn their species-specific songs from conspecifics, they were trained to sing folk melodies whistled to them. One bullfinch learned a 45-note tune from a human tutor and sang it in transposition (i.e. at a different pitch level), indicating exceptional vocal learning and relative pitch processing skills, also incorporating appropriate rhythm. Other bullfinches alternated parts with the human tutor, as in antiphonal singing, indicating that they anticipated as well as followed the notes of a learned melody. Experiments such as these build on the natural abilities of animals, as revealed by field research, productively extending them to controlled contexts.

Snowball, the sulfur-crested cockatoo (*Cacatua galerita eleonora*) whose dance video became a YouTube sensation, helped renew scientific interest in entrainment in non-human species. Systematic study revealed that Snowball could synchronize his movements to the beat of music and adjust his rate of movement to changes in tempo [98], challenging the notion that entrainment is uniquely human. The authors suggested, moreover, that such entrainment might be evident in other species of vocal learners. In fact, a study of YouTube videos featuring animal ‘dancing’ provided confirmation of entrainment to music in vocal learning species but not in other species (e.g. dogs [12]). Another consistent finding was successful training of a budgerigar (*Melopsittacus undulates*) to tap along with a beat [99].

That only vocal learners have the capacity for entrainment seems reasonable, given that the three avian groups in which vocal learning has evolved independently have similar functional neural pathways that are not shared with non-vocal learners, and are comparable to humans [100]. That is, they have a direct connection between auditory perception areas and motor areas [101]. Entrainment may necessitate this kind of neural architecture [94]. At the same time, Schachner *et al.* [12] found evidence for entrainment in only one of the avian vocal learning subgroups, the parrot species, and not in songbirds. The only non-parrot species in which entrainment has been detected to date is elephants. Although elephants show evidence of vocal learning [11] their vocal learning mechanism is unknown, but is likely to differ from that of parrots. A compelling recent study showed that a California sea lion (*Zalophus californianus*) could also be trained to synchronize with a beat, and then spontaneously generalized to music [102]. This species is not thought to be a vocal learner, although some other pinnipeds are vocal learners [11]. It is possible that sea lions have vocal learning abilities that are as yet unknown. However, it could also be that the ability to synchronize with a beat only requires part of what is required for vocal learning, or even that entrainment abilities can occur without any of the components for vocal learning. Another recent study showed that a chimpanzee, one of the closest non-vocal learning relatives of humans, spontaneously entrained to a beat while completing a motor tapping task [103]. Clearly, the proposed connection between vocal learning and entrainment [94] requires further research with species that are not vocal learners.

If entrainment is defined more broadly, it could include many non-vocal learning species such as several species of fireflies synchronizing flashing with one another [104], and several species of frogs [105] and katydids [106] synchronizing their chorusing. Identifying a pulse and locking in phase with it is a simpler task than detecting and entraining to a beat within a stream of music, where the beat is not always marked with an acoustic event, and other acoustic events are present between beats (see [94] for review). Understanding the range of natural abilities related to entrainment could clarify what is relevant for musicality.

There are other potentially productive means of studying the precursors of musicality in non-human species. One approach is to search for music-like features in animal vocalizations. For example, Araya-Salas [107] examined whether the pitch ratios created by adjacent notes of the song of nightingale wrens (*Microcerculus philomela*) conform to harmonic pitch ratios. From 243 comparisons, only six were significantly close to harmonic pitch ratios, suggesting no consistent use of harmonic pitch ratios. Another approach builds on the studies by Hartshorne [108] and others in seeking ‘aesthetic’ features in natural birdsong that might have arousing or emotive consequences, as music does for human listeners. This notion was met with considerable scepticism, but Rothenberg *et al.* [109] posed a similar question with thrush nightingales (*Luscinia luscinia*). According to their analysis, the songs of thrush nightingales have similar patterns of tension and resolution to those of music, which create expectation and anticipation in human listeners. If a certain level of familiarity and novelty is valued across species that produce complex songs, this could lead to insights into the origins of our motivation for music.

Another route to discovering music-like behaviours in non-human species is to make predictions from the natural behaviour of humans. For example, humans generalize across timbres, recognizing a melody, regardless of the instrument on which it is played. In most cases, it makes sense not to generalize across timbres. Different spectral information can change the meaning of vocalizations not only in human speech with different vowels, but also in animal vocalizations [110]. A study species that may be more fruitful for timbre generalization research would be a species that mimics the vocalizations of other species. For satin bowerbirds (*Ptilonorhynchus violaceus*) female preference for mates and male mate success may depend on the accuracy with which males imitate heterospecific vocalizations [111]. If the mimetic accuracy is what is important, and not simply how well-learned a song is (as has been shown to be important in other species, [112]), female bowerbirds would need to assess the original heterospecific vocalization, and the conspecific imitation, in a way that is similar to a human evaluating a singer's performance in comparison with a pianist. She would need to be able to distinguish the two, but also generalize between them in the sense that she is aware that they are meant to be the same thing. In short, reflecting on natural human and non-human behaviours that are musically relevant can provide ideas about species and abilities that offer promising directions for comparative study.

### (c) Integrating natural and artificial studies

Naturalistic studies have revealed important abilities and questions related to the biological basis of music such as vocal learning and entrainment. They have also suggested new directions for laboratory research.

Laboratory studies often reveal abilities that are not used by non-human species under natural conditions. Knowledge of the underlying capacity for those abilities can contribute to an understanding of their evolutionary, developmental and physiological foundations. The capacity for a particular ability, even if it is unrealized in nature, may arise from the evolutionary history of the species. Identifying the requirements of such abilities and their evolutionary pressures may be facilitated by studying the limits of these abilities.

There is increasing research on aspects of musicality in various non-human species, but it is rare to find naturalistic studies of musically relevant abilities and studies of the limits of those abilities in the same species. A rare but productive example of a combined approach involves the chickadee, which has been the subject of extensive field and laboratory research. This blend of research methods made it possible to understand the relative pitch processing skills of this species [69,86–89]. Comparably important insights might arise from increased field research with species that have received extensive experimental study and increased laboratory research with species whose natural behaviours have been well documented.

## 5. Conclusion

At present, there is limited laboratory research on the components of musicality in non-human species although there is increasing interest in this domain, so considerable expansion of this research direction is likely. As noted, traditional laboratory studies and naturalistic studies can provide equally important and complementary insights into musically relevant skills. One example noted earlier was finer pitch [46–49] and temporal [13] resolution in birds than in mammals, which emerged from laboratory studies, and vocal learning and entrainment abilities in some bird and mammal species [11–12,94], which emerged from naturalistic studies.

Currently, vocal learning and entrainment are the principal focus of research on musically related behaviours and their underpinnings in non-human species. There are other potentially productive questions that could be pursued. For example, what kinds of behaviour require relative pitch preferences like those present in chickadees [89]? What kinds of behaviour require timbre generalization like that observed in zebra finches [36–37]? Why is auditory grouping relevant in some species? One way forward is to search for relevant natural behaviours in less studied species and to examine the natural behaviours of species commonly studied in the laboratory.

## Footnotes

1 For definitions of musical terms, please see the glossary, table 1.
